# Sonothrombolysis Augments Reperfusion in ST-Elevation Myocardial Infarction With Primary Percutaneous Coronary Intervention: Insights From the SONOSTEMI Study

**DOI:** 10.1016/j.cjco.2022.03.004

**Published:** 2022-03-12

**Authors:** Kevin R. Bainey, Ahmed Abulhamayel, Amir Aziz, Harald Becher

**Affiliations:** a Canadian VIGOUR Centre, University of Alberta, Edmonton, Alberta, Canada; b Mazankowski Alberta Heart Institute, University of Alberta, Edmonton, Alberta, Canada

## Abstract

Reperfusion injury is common following primary percutaneous coronary intervention (PCI) in ST-elevation myocardial infarction. In a prospective Canadian single-arm study of 15patients, the use of myocardial contrast echocardiography with high mechanical index ultrasound impulses (sonothrombolysis) initiated prior to primary PCI resulted in 7 patients with pre-PCI thrombolysis in myocardial infarction-2/3 flow (46.7%). Following reperfusion, all 15 patients had thrombolysis in myocardial infarction-3 flow, and 14 patients achieved ST-segment resolution ≥ 50% at 30 minutes post-PCI (93.3%). At 90 days, 12 patients had normal left ventricular ejection fraction ≥ 50% (80.0%). Our results demonstrate the feasibility of a novel technique to enhance reperfusion in ST-elevation myocardial infarction and provide a rationale for a randomized Canadian study.

## Résumé

La lésion de reperfusion est fréquente après l’intervention coronarienne percutanée (ICP) primaire chez les patients atteints d’un infarctus du myocarde avec élévation du segment ST. Dans une étude prospective canadienne, à volet unique, auprès de 15 patients, l’utilisation de l’échocardiographie myocardique de contraste par des impulsions ultrasonores à indice mécanique élevé (sonothrombolyse) amorcée avant l’ICP primaire s’est traduite par sept patients qui ont eu une thrombolyse pré-ICP de l’infarctus du myocarde de flux de grade 2/3 (46,7 %). Après la reperfusion, les 15 patients ont subi une thrombolyse de l’infarctus du myocarde de flux de grade 3, et 14 patients ont eu une résolution du segment ST ≥ 50 % 30 minutes après l’ICP (93,3 %). Après 90 jours, 12 patients ont eu une fraction d’éjection ventriculaire gauche normale ≥ 50 % (80,0 %). Nos résultats démontrent la faisabilité d’une nouvelle technique pour améliorer la reperfusion des infarctus du myocarde avec élévation du segment ST et justifient la réalisation d’une étude canadienne à répartition aléatoire.

Although prompt reperfusion with primary percutaneous coronary intervention (pPCI) improves clinical outcomes in ST-elevation myocardial infarction (STEMI), abrupt recanalization often leads to reperfusion injury and microvascular dysfunction. In the first North American study, we evaluated the effects of sonothrombolysis on epicardial patency, myocardial perfusion, and left ventricular performance in STEMI.

## Methods

**Sono**thrombolysis in **ST**-**E**levation **M**yocardial **I**nfarction (SONOSTEMI) was a prospective, single-centre, single-arm study of subjects presenting within 6 hours of chest pain and found to have a STEMI requiring pPCI (NCT03092089). Following consent, sonothrombolysis was administered as early as possible prior to pPCI as previously described.^1^ Briefly, an intravenous infusion of Definity microbubble ultrasound contrast (Lantheus Medical Imaging, North Billerica, MA) was administered (1-2 mL/min) prior to PCI and was continued post-PCI for a total of 30 minutes. Myocardial contrast echocardiography was performed (EPIQ, Philips-X5-1 transducer, Koninklijke Philips N.V., Amsterdam, the Netherlands) with presets for myocardial perfusion imaging. Loops of 15 cardiac cycles were recorded using low mechanical index ultrasound with a “flash” delivered after the second cardiac cycle of the loop. The flash is a short impulse of high mechanical index ultrasound, which is transmitted to destroy contrast microbubbles. The intervals between the high mechanical index ultrasound impulses varied from 5 to 15 seconds depending on the time required for myocardial contrast replenishment.

## Results

**Figure 1:**
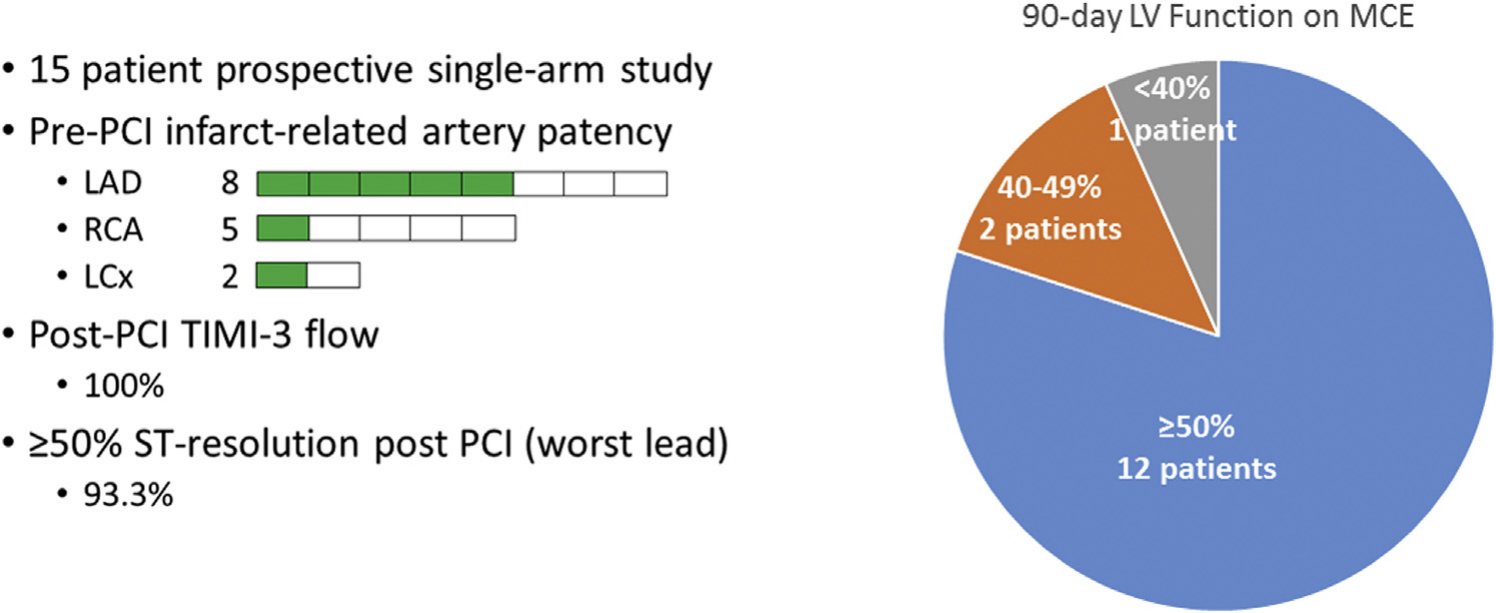
Summary of the findings from the SONOthrombolysis in ST-Elevation Myocardial Infarction (SONOSTEMI) study. LAD, left anterior descending artery; LCx, left circumflex artery; LV, left ventricular; MCE, myocardial contrast echocardiography; PCI, percutaneous coronary intervention; RCA, right coronary artery; TIMI, thrombolysis in myocardial infarction.

Between August 2017 and June 2019, 15 subjects were enrolled (53.3% anterior STEMI) with a mean age of 61.8 ± 8.0 years, and 26.7% were female. Table 1 provides a summary of the baseline and procedural characteristics. The average infusion time for contrast agent was 12.5 ± 9.3 minutes pre-PCI, and 23.3 ± 4.1 minutes post-PCI. Immediately prior to coronary angiography, 11 of 15 patients had a pre-PCI electrocardiogram performed, of which 2 patients had ≥ 50% ST-segment resolution (worst lead) pre-PCI (18.1%). At coronary angiography, 7 patients had a patent epicardial vessel with pre-PCI thrombolysis in myocardial infarction (TIMI)-2/3 flow (46.7%). Of the 8 culprit left anterior descending artery infarcts, 5 were patent; of the 5 right coronary artery infarcts, 1 was patent; of the 2 left circumflex artery infarcts, 1 was patent. Following reperfusion with pPCI, all 15 patients had TIMI-3 flow, and 14 patients achieved ST-segment resolution ≥ 50% (worst lead) ∼30 minutes post-PCI (93.3%; Canadian VIGOUR Centre electrocardiogram core laboratory analysis, University of Alberta, Edmonton). Myocardial contrast echocardiography was performed at baseline (pre-PCI), at day-1 (immediately post PCI), at discharge (∼3 days post-PCI,) and at 90-day follow-up. Improvement in left ventricular ejection fraction was observed (51.5% ± 11.6%, 54.5% ± 12.2%, 54.6% ± 9.9%, 54.9% ± 10.6%, respectively). Wall motion score index and myocardial perfusion score index were reduced (wall motion score index: 1.8 ± 0.3, 1.6 ± 0.3, 1.5 ± 0.3, 1.4 ± 0.4, respectively; myocardial perfusion index: 1.7 ± 0.3, 1.4 ± 0.3, 1.3 ± 0.2, 1.2 ± 0.2, respectively). At 90-days, 12 patients had normal left ventricular ejection fraction ≥ 50% (80.0%; Fig. 1).

**Table 1:** Baseline and procedural characteristics of the **Sono**thrombolysis in **ST**-**E**levation **M**yocardial **I**nfarction (SONOSTEMI) study patients (n = 15)

Baseline characteristics	
Age, y	61.8 ± 8.0
Male sex	73.3
Hypertension	53.3
Diabetes	6.7
Hypercholesterolemia	46.6
Current/former smoker	33.3
Prior MI	20.0
Procedural characteristics	
Anterior MI	53.3
Ischemic time, min	172.0 ± 71.4
FMC-device time, min	76.5 ± 64.8
Infarct-related artery territory	
Left anterior descending artery	53.3
Left circumflex artery	13.3
Right coronary artery	33.3

Values are %, or mean ± standard deviation.

FMC, first medical contact; MI, myocardial infarction.

## Discussion

Although this novel technique has been tested in STEMI patients in São Paulo, Brazil,^2^ this is the first proof-of-concept North American study of sonothrombolysis in the setting of timely pPCI with short ischemic times. In this context, sonothrombolysis is feasible when it is initiated prior to coronary intervention, as we found no appreciable delay to pPCI, with an average of 76.5 minutes from first medical contact to device time, well below the recommended guideline metric of 90 minutes.^3^ Almost one-half of participants had pre-PCI TIMI-2/3 flow, well above expected spontaneous reperfusion rates.^4^ We observed a higher rate of pre-PCI TIMI-2/3 flow in those with a left anterior descending artery infarct, likely due to the anatomic position of the left anterior descending artery. We observed a high rate of ≥ 50% ST-segment resolution post-PCI (a marker of myocardial perfusion)—greater than expected in STEMI,^5^ and possibly a reflection of enhanced microvascular function with sonothrombolysis. Finally, the enhancement in left ventricular performance at 3 months is notable given the degree of improvement in wall motion score index and left ventricular function when compared to remodeling findings in STEMI.^6^ Our results are notable and provide further data to support this novel technique in augmenting reperfusion in STEMI, both at the epicardial and myocardial level, which we believe translates to enhanced left ventricular performance. Still, these findings are hypothesis-generating and require confirmation in a Canadian randomized trial enrolling subjects with short ischemic times.
